# Development of a Quartz-Based Photo-Mobile Polymer Film for Controlled Motion Triggered by Light or Heat

**DOI:** 10.3390/ma16083046

**Published:** 2023-04-12

**Authors:** Riccardo Castagna, Cristiano Riminesi, Maria Savina Pianesi, Simona Sabbatini, Andrea Di Donato, Gautam Singh, Oriano Francescangeli, Emma Cantisani, Paolo Castellini, Daniele Eugenio Lucchetta

**Affiliations:** 1 URT-CNR@UNICAM, Photonic Materials Laboratory, Consiglio Nazionale delle Ricerche (CNR), Università di Camerino (UNICAM), Ex-Carmelitane, Via Sant’Agostino, 1, 62032 Camerino, MC, Italy; cristiano.riminesi@cnr.it; 2 CNR, Institute of Heritage Science, Via Madonna del Piano, 10, 50019 Sesto Fiorentino, FI, Italy; 3 Delta s.r.l., Via G. Deledda, 3, 62010 Montecassiano, MC, Italy; savina.pianesi@pladostelma.com; 4 Dip. SIMAU, Università Politecnica delle Marche, Via Brecce Bianche, 60131 Ancona, AN, Italy; 5 Dip. DII, Università Politecnica delle Marche, Via Brecce Bianche, 60131 Ancona, AN, Italy; 6 Department of Applied Physics, Amity Institute of Applied Sciences, Amity University, Noida 201313, Uttar Pradesh, India; 7 Dip. DIISM, Università Politecnica delle Marche, Via Brecce Bianche, 60131 Ancona, AN, Italy; 8 Optoacoustic Lab, Dip. SIMAU, Università Politecnica delle Marche, Via Brecce Bianche, 60131 Ancona, AN, Italy

**Keywords:** photo-mobile polymer, sensors, contact-less actuator, green polymers, green energy, energy harvesting

## Abstract

We have developed a photo-mobile polymer film, that combines organic and inorganic materials, to allow for controlled motion that can be triggered by light or heat. Our film is made using recycled quartz and consists of two layers: a multi-acrylate polymer layer and a layer containing oxidized 4-amino-phenol and N-Vinyl-1-Pyrrolidinone. The use of quartz in our film also gives it a high temperature resistance of at least 350 °C. When exposed to heat, the film moves in a direction that is independent of the heat source, due to its asymmetrical design. Once the heat source is removed, the film returns to its original position. ATR-FTIR measurements confirm this asymmetrical configuration. This technology may have potential applications in energy harvesting, due to the piezoelectric properties of quartz.

## 1. Introduction

The field of composite materials research is extensive and encompasses several relevant applications in science and technology [1,2,3,4,5,6,7]. As a specific type of composite material, photo-responsive polymers have the ability to change physical, chemical, or optical properties when exposed to light [8,9]. Depending on the intensity and type of light source, these polymers can undergo reversible or irreversible changes in their molecular structures, colors, or shapes. These properties make photo-responsive polymers highly attractive for various applications, including optical data storage, smart windows, and sensors. Photo-mobile polymers (PMPs) are particular kinds of photo-responsive polymers having motion which can be induced without contact and controlled by an external electromagnetic field, typically in the form of a light source [10,11,12,13,14,15]. Research on PMPs is rapidly accelerating due to their potential applications in energy harvesting, light-controlled actuators, and light-induced dynamic holography [13,14,16,17,18]. On the other hand, thermo-responsive polymers [19,20] can change their properties in response to temperature changes. In recent years, advances in materials science have led to the development of polymer materials that exhibit both photo- and thermo-responsive properties. These dual-responsive polymers can change their properties in response to both light and/or heat, making them highly versatile and suitable for a variety of applications. Moreover, there is growing competition to develop new technologies that utilize recycled materials, which is a significant and ongoing challenge. To address this issue, we propose recycling waste from industrial processes to create energy-generating materials in the future. Specifically, we suggest incorporating quartz, a material known for its piezoelectric properties, into a photo-mobile composite polymer system to produce thermally-induced motion of the photo-mobile polymer film. The direction of the movement is not influenced by the direction of the heating source, and the motion can be controlled by a heating source or an UV-visible-near infrared (UV, VIS-NIR) source. Additionally, we demonstrate that this type of photo-mobile composite polymer has high temperature resistance, making it possible to induce motion using a nearby welder or by means of direct contact with the polymer surface using indium–tin oxide [21,22].

## 2. Materials and Methods

### 2.1. Materials

Phenyl-bis(2,4,6-trimethylbenzoyl) phosphine oxide (I819), 4-aminophenol (4-AP), N-Vinyl-1-Pyrrolidonone (NVP), dipentaerythrythol-hydroxy-penta/hexa-acrylate (DPHPA) were acquired from Merck, Darmstadt, Germany. A mixture containing Titanium(IV) oxide (TiO${}_{2}$) and quartz (SiO${}_{2}$) was acquired from Delta-materials (DeltaMAT) by Delta s.r.l., Montelupone, MC, Italy.

### 2.2. Preparation of the t-PMP-Mixture

In a small bottle, 1 mmol of 4-AP and NVP (≈5 mmol) were subjected to oxidation in the presence of a DeltaMAT grain mixture, containing ≈0.20 mmol of TiO${}_{2}$. The system was left in aerobic conditions in the dark for 10 days. The resulting red liquid phase was extracted. Separately, 1 mmol of DPHPA was mixed with 0.14 mmol of I819 for ≈3 h in the dark. Finally, all components were combined and left to react in darkness at room temperature for 7 days.

### 2.3. Preparation of the t-PMP-Film

The procedure required the introduction of the PMP mixture into a cell, made up of two glass slides separated by 50 μm thick mylar stripes, using capillary action. The cell was then subjected to UV-A irradiation for approximately 30 min. Subsequently, the cell was opened, and the resulting t-PMP film peeled off. For our experiments, t-PMP films weighing approximately 1 mg were utilized.

### 2.4. Set-Up

The experimental setup was mainly based on a Basler CCD camera that was placed in front of the t-PMP film, which was, in turn, placed in front of a ruler. This system was used to record the movements, from which graphs were extracted, in order to have an exact record of the motion of the object in the proximity of the heating source. The heating source was a soldering iron at c.a. 5 mm from the basis of the t-PMP-stripe. The t-PMP was around 0.5 × 0.8 cm${}^{2}$ [23].

### 2.5. ATR-FTIR Measurements

IR spectra were acquired in reflectance mode by means of a Perkin Elmer Spectrum GX1 spectrometer, using an ATR accessory equipped with a ZnSe crystal (Spectrum software 10.4.0, Perkin-Elmer, Waltham, MA, USA). The spectral range was 4000–400 cm${}^{-1}$ (spectral resolution of 4 cm${}^{-1}$ and 32 scans for each spectrum). The sample was deposited directly onto the ZnSe crystal without requiring any preparation. Three IR spectra were acquired and the average asborbance spectrum was calculated. The background spectrum was performed on the clean crystal under the same conditions before each acquisition. IR spectra were converted to absorbance and were vector normalized.

### 2.6. X-ray Measurements

An X’Pert Pro PANalytical diffractometer was equipped with an X’Celerator detector, with a Cu X-ray tube (λ= 1.54 A) and a Ni-filtered Cu–Kα radiation source. The X-ray tube was operated at 40 kV and 30 mA. The diffraction patterns were collected from 3–70${}^{\circ }$ 2θ with a step size of 0.02${}^{\circ }$. A zero-background sample stage was used. For phase identification of the XRD results, the Powder Diffraction (PDF) database from the International Centre for Diffraction Data (ICCD) was used.

## 3. Results and Discussion

**Figure 1:**
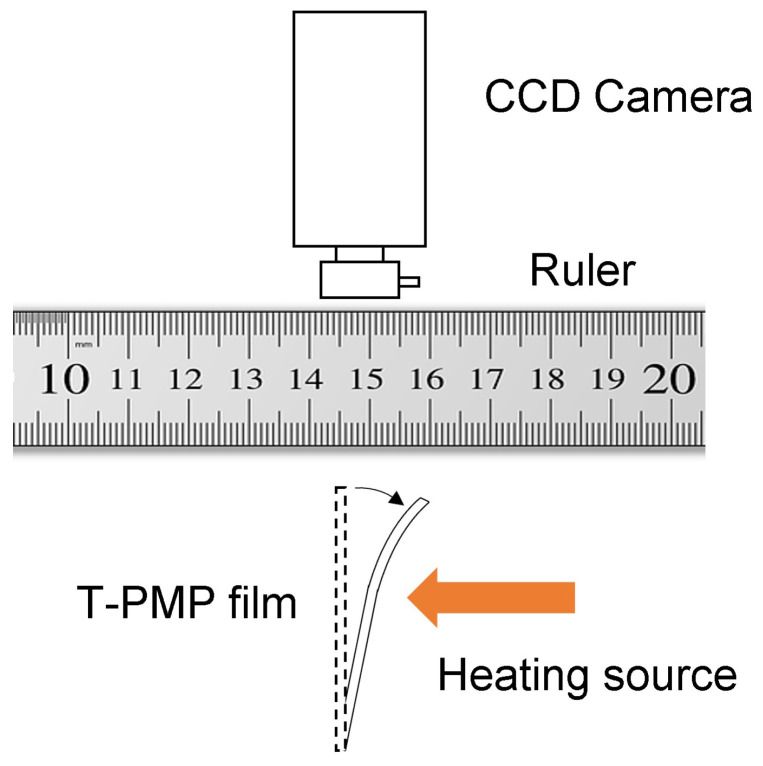
The experimental setup used to measure the behavior of the t-PMP-film under, and after, heat radiation. A CCD camera was used to record motion.

We conducted measurements on a rectangular-shaped t-PMP film (≈0.5 × 0.8 cm${}^{2}$). The material we used was similar to that described in our previous publications, as referenced in [16], with the exception that we substituted TiO${}_{2}$ for PbO${}_{2}$ and added SiO${}_{2}$ (alpha-quartz and cristobalite) to the typical PMP mixture (see Materials Section). The PMP material works primarily through the oxidation of NVP and 4-AP, which acts as the driving force of the system when exposed to electromagnetic radiation [16,17]. This oxidation enables the compound to dissolve in the system, while also causing a sudden increase in the system’s absorbance. As we previously noted, the material is more viscous than the starting solvent (NVP) and repels light [17]. We used materials derived from industrial waste to induce the oxidation, specifically waste from the sink industry, which we had previously characterized and which we refer to as “DeltaMAT” in this document. DeltaMAT is a mixture of micro-grains containing compounds identified through XRD analysis. The mixture primarily comprises alpha-quartz and cristobalite, with the addition of TiO${}_{2}$ nanoparticles that drive the oxidation of 4-AP in NVP. TiO${}_{2}$ is responsible for the system’s oxidation, and the mixture is left under normal conditions until it turns dark red. The mixture is then placed between two 1 mm thick glass slides, with an inter-glass distance of 50 μm, and exposed to a UV-A lamp for approximately 30 min, resulting in photopolymerization. The acrylate is polymerized while the oxidized 4-AP-doped NVP is repelled under irradiation, resulting in an asymmetric bilayered film, due to gravity and light-repulsion. The quartz grains are located on the lower side of the cell, farther from the UV-A source, as evidenced by a visual inspection of the film’s smooth and rough sides. After removing the sample from the glass sandwich, its behavior under heat was observed (refer to the Scheme of Figure 1).

**Figure 2:**
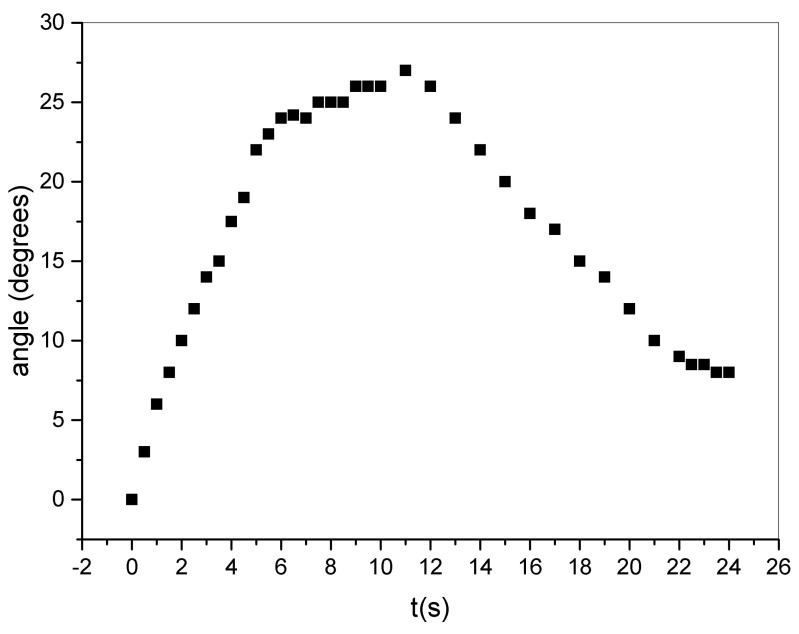
Behavior of the t-PMP under heat radiation. Bending (${}^{\circ }$) vs. time (s).

The motion of the film under illumination is reported in Figure 2.

**Figure 3:**
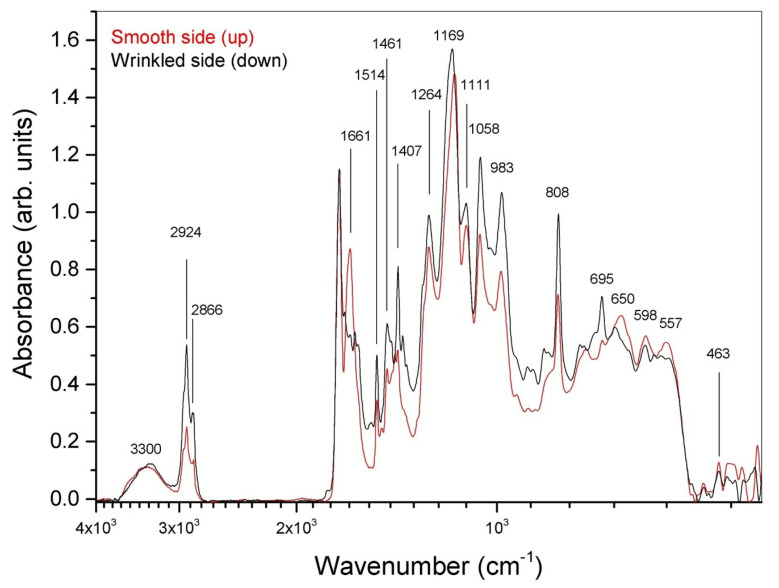
ATR-FTIR measurements operated on the upper and lower faces of the t-PMP film.

The sample exhibited high resistance to heating, allowing the tip of a welder to be brought close to its surface without damage. To induce motion, the sample was clamped and heated using a welder with a measured temperature of approximately 350 °C at the tip, positioned about 2 mm away from the sample on both sides. The direction of motion was independent of the direction of the heat source. The overall response of the sample was recorded over a 24 s period (see Figure 2). The sample was exposed to the heating source for ≈11 s. During the first 6s the bending rate reached its maximum. After that, the bending rate decreased in a few seconds and gradually approached zero during the plateau stage. Once the heat source was removed (arrow in Figure 2), the sample gradually returned to its original position. As we stated above, the direction of bending was not influenced by the direction of the heat source. This observation provided indirect evidence that the film was asymmetrical. To further confirm this hypothesis, we conducted ATR-FTIR measurements on both sides of the t-PMP film. By comparing the ATR-FTIR spectra obtained from the upper and lower faces of the t-PMP film (as shown in Figure 3), we observed a clear difference between the two sides. However, we are unable to determine the relative abundance of quartz on one face compared to the other. Specifically, we hypothesized that there was a higher degree of polymerization on the upper face of the t-PMP film, based on the differences in the 809 cm${}^{-1}$ peaks. The peak at 1660 cm${}^{-1}$, which appeared in the smoothed region, might indicate copolymerization between acrylate and NVP. Regardless of the peak attribution, the ATR-FTIR analysis confirmed that the two sides of the t-PMP film were different from each other.

**Figure 4:**
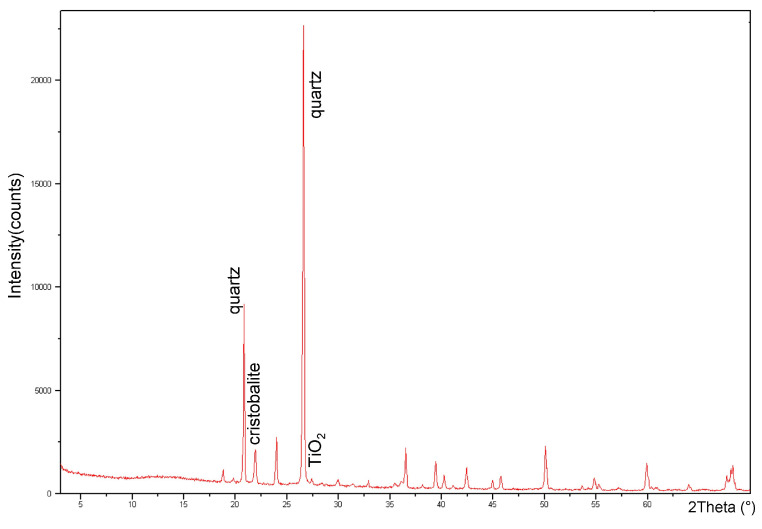
XRD of the starting inorganic deltamat materials.

We here remark that the starting inorganic mixture included alpha-quartz, cristobalite and TiO${}_{2}$ nanoparticles, as reported in the XRD graph in Figure 4. However, at present, we are not able to markedly discriminate the presence of the inorganic material on the two faces of the film, even if the peaks at 1060 cm${}^{-1}$ and 985 cm${}^{-1}$ could be attributed to quartz.

**Figure 5:**
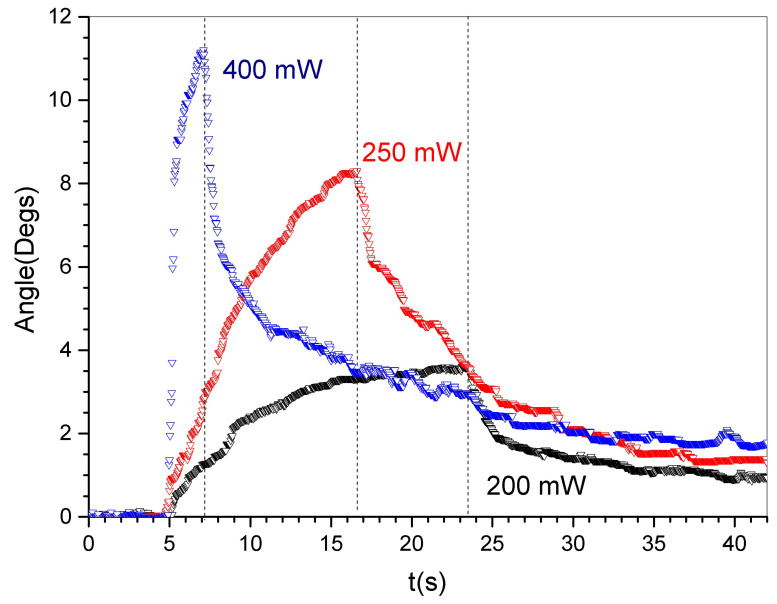
Behavior of the t-PMP under light irradiation: bending angle (${}^{\circ }$) vs. time (s).

As depicted in Figure 5, the t-PMP-film maintained its ability to move when exposed to light with a wavelength of 532 nm, even at relatively high power/intensity levels (P = 200, 250, and 400 mW, with a spot diameter of 3 mm). The sample began to burn when exposed to powers higher than 400 mW. As the irradiation power increased from 200 to 400 mW, the film achieved higher bending angles in a shorter amount of time (from approximately 18.5 s and 3.5${}^{\circ }$ to approximately 2.0 s and 11.5${}^{\circ }$, respectively).

**Figure 6:**
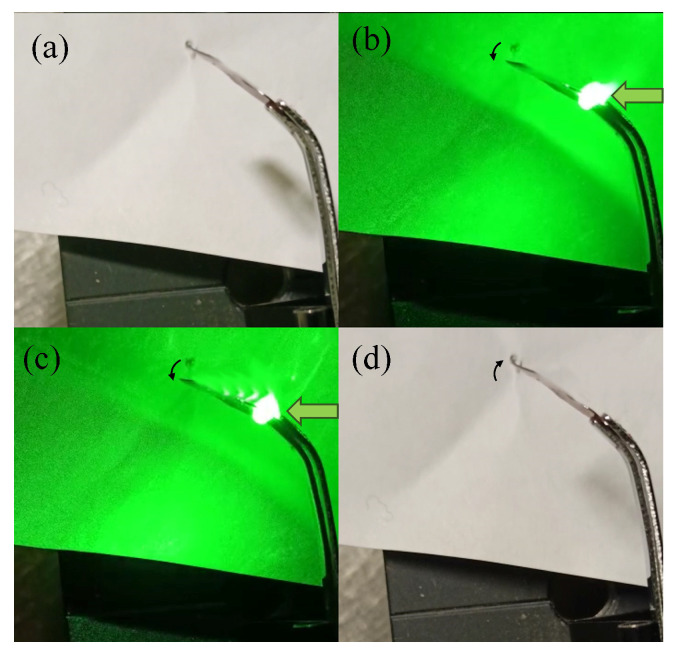
The frames show how the film bent over time under different conditions: (**a**) before being exposed to irradiation, (**b**) after 1 s of irradiation, (**c**) after 2 s of irradiation, and (**d**) after complete relaxation. The irradiation source had a power of 400 mW. The black arrows indicate the direction of movement, while the green arrows represent the direction of the irradiation source.

When exposed to 400 mW of irradiation, the t-PMP film reached its maximum bending angle in approximately 2 s. Once the irradiation ended, the film began to return to its original position. In the beginning, the motion was fast, but complete restoration took several minutes. Figure 6 depicts frames that represent the film’s position at different times.

**Figure 7:**
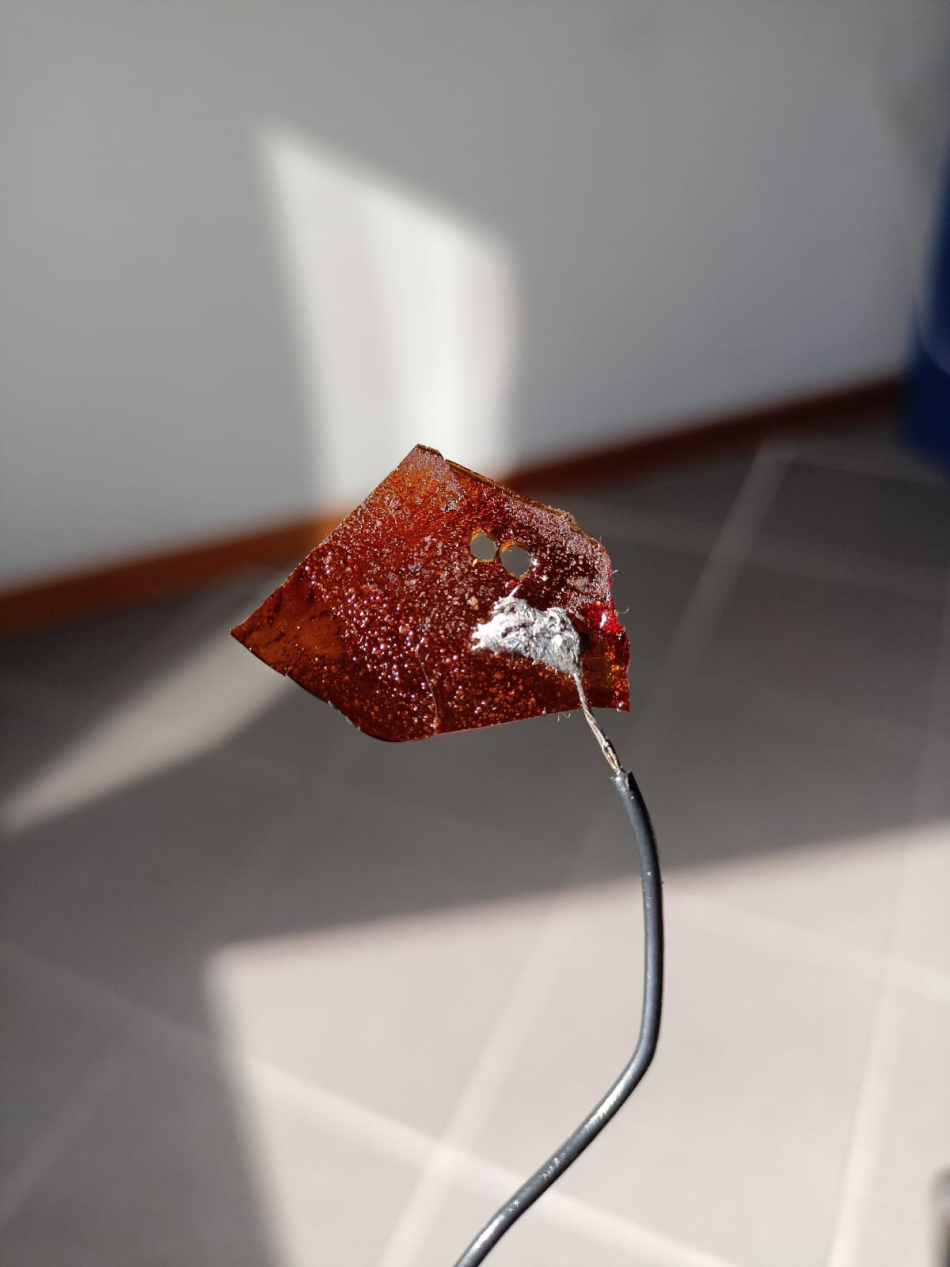
A copper wire soldered with a 60/40 Sn-Pb paste on the quartz-rich part of the t-PMP-film.

Furthermore, the film exhibited high-temperature resistance (see Figure 7), as demonstrated by its ability to allow direct soldering of a copper wire onto its surface. This property makes it suitable for the construction of systems that require high temperatures in direct contact with the film.

## 4. Conclusions

In brief, a novel hybrid organic/inorganic t-PMP film was created, that includes SiO${}_{2}$ (quartz/cristobalite). This film can be activated by either heat or light to induce movement, and upon removal of the heat or light source, the film returns to its original position. Furthermore, this material can endure high temperatures of at least 350 ${}^{\circ }$C and can be soldered with a common tin–lead welding mixture into a copper wire. The film’s capacity to respond to heat, together with the piezoelectric properties of quartz, suggest promising future applications in 3D dynamic displays and energy harvesting.

## Data Availability

Data are available from the authors under reasonable request.
